# A Simplified Population-Level Landscape Model Identifying Ecological Risk Drivers of Pesticide Applications, Part One: Case Study for Large Herbivorous Mammals

**DOI:** 10.3390/ijerph18157720

**Published:** 2021-07-21

**Authors:** David Tarazona, Guillermo Tarazona, Jose V. Tarazona

**Affiliations:** 1 Independent Researcher, 28009 Madrid, Spain; davidtala@gmail.com; 2 PharmaMar, Colmenar Viejo, 28770 Madrid, Spain; gpr.tarazona@gmail.com; 3 Scientific Committee and Emerging Risks Unit, European Food Safety Authority, 43126 Parma, Italy

**Keywords:** pesticides, landscape risk assessment, population model, rabbit, hare

## Abstract

Environmental risk assessment is a key process for the authorization of pesticides, and is subjected to continuous challenges and updates. Current approaches are based on standard scenarios and independent substance-crop assessments. This arrangement does not address the complexity of agricultural ecosystems with mammals feeding on different crops. This work presents a simplified model for regulatory use addressing landscape variability, co-exposure to several pesticides, and predicting the effect on population abundance. The focus is on terrestrial vertebrates and the aim is the identification of the key risk drivers impacting on mid-term population dynamics. The model is parameterized for EU assessments according to the European Food Safety Authority (EFSA) Guidance Document, but can be adapted to other regulatory schemes. The conceptual approach includes two modules: (a) the species population dynamics, and (b) the population impact of pesticide exposure. Population dynamics is modelled through daily survival and seasonal reproductions rates; which are modified in case of pesticide exposure. All variables, parameters, and functions can be modified. The model has been calibrated with ecological data for wild rabbits and brown hares and tested for two herbicides, glyphosate and bromoxynil, using validated toxicity data extracted from EFSA assessments. Results demonstrate that the information available for a regulatory assessment, according to current EU information requirements, is sufficient for predicting the impact and possible consequences at population dynamic levels. The model confirms that agroecological parameters play a key role when assessing the effect of pesticide exposure on population abundance. The integration of laboratory toxicity studies with this simplified landscape model allows for the identification of conditions leading to population vulnerability or resilience. An Annex includes a detailed assessment of the model characteristics according to the EFSA scheme on Good Modelling Practice.

## 1. Introduction

Environmental risk assessment is a key process for the authorization of pesticides, subjected to continuous challenges and updates [1]. In the regulatory context, different agencies have developed models and scenarios for pre-marketing and re-evaluation assessments. The proposed pesticide uses patterns, named “Good Agricultural Practices (GAPs)”, which are tested according to these models and scenarios, checking whether or not the estimated level of risk is in line with the protection goals defined by risk managers. Exceeding the accepted level of risk means non-approval or mandatory risk mitigation options, such as maximum application dose, limited number of applications within the season, untreated buffer zones, etc. In the EU system, the protection goals are generic, although the European Food Safety Authority (EFSA) has suggested the development of specific protection goals [2], based on the concept of ecosystem services. For terrestrial mammals, current protection goals include acute lethality and markers for population level effects. These are described in the legislation, based on a threshold approach based on Reference Points (also named Points of Departure) extracted from experimental studies and uncertainty factors to cover interspecies variability, and are implemented in a specific guidance document [3].

Standard scenarios are frequently used in the regulatory context, particularly for assessments related to pre-marketing authorizations. The EFSA Guidance Document on birds and mammals [3] details those used for the EU assessments. The use of standard scenarios offers clear benefits, including harmonization, equal treatment, and certainty for registrants. However, there are also significant limitations, in particular, worst-case assumptions are needed for the lower tiers, not accounting for the real variability. Although this approach offers a high level of protection when low risk is identified at the lower tiers; when higher tier refinement is needed, the legislation does not provide specific advice on the actual level of effects and time for recovery considered acceptable by risk managers.

During the last decade, ecological models have been suggested as the alternative for more informative assessments [4]. Toxicokinetic and toxicodynamic modelling allows the escalation of the effects observed in the lab to consequences on the population. EFSA has provided recommendations for using these models in regulatory risk assessments focusing on aquatic species [5], and several authors have also explored their application to mammalian risk assessments [6]. In addition, landscape-based models cover the expected variability in habitats and agricultural conditions, addressing not only the variability in exposure but also the expected consequences of toxic effects on individuals at population level; and may be expanded to address differences in resilience and recovery. The Animal, Landscape, and Man Simulation System (ALMaSS) model [7,8,9] is an excellent example of the implementation of realistic landscape conditions and local land management in GIS-based models, to be used for pesticides risk assessment [10].

Despite the efforts and significant developments, the incorporation of ecological modelling in pesticide risk assessment is still limited to a few cases at a high tier level. Validation in the field is very complex as pesticides are just one of the factors affecting population dynamics, and regulatory acceptance was limited by the absence of specific guidance on regulatory needs. Several regulatory agencies have tried to address this challenge, developing recommendations and regulatory frameworks. In Europe, the responsible Scientific Panel of EFSA has developed general advice on good modelling practices with criteria to be followed by model developers for regulatory pesticide risk assessment [11]. In the US, the Environmental Protection Agency (USEPA) has developed a framework for facilitating the regulatory use of ecological models [12]. We have explored and combined both approaches, developing a simplified ecological model for assessing the risk of pesticides to terrestrial vertebrates under realistic conditions. The aim was to develop a model with regulatory focus, implementing regulatory problem formulations and supporting fit-for-purpose pre-marketed risk assessment [13]. In addition, the model can be used for estimating the contribution of pesticides in environmental impact assessments; implementing the conceptual framework proposed in a previous publication [14]; and addressing some of the research priorities for a sustainable environmental quality assessment [15].

Recent EFSA assessments (all available at www.efsa.europa.eu, (accessed on 13 December 2020)) have identified possible concerns to herbivorous mammals for the herbicides glyphosate and bromoxinyl. Glyphosate is a broad-spectrum systemic herbicide and crop desiccant, while bromoxynil and its esters are widely used as selective contact herbicides for post-emergent control of broad-leaved weed. These herbicides have been used for building up a proof-of-concept case-study for “context of use qualification” of the model.

## 2. Materials and Methods

### 2.1. Model Description

Appendix A provides a detailed description following EFSA good modelling practices [11]. A summary is provided below. In addition to good modelling practices for pesticide risk assessment [11,16]; additional requirements were considered for ensuring the model capability for supporting environmental management decisions [17,18].

#### 2.1.1. Model Conceptualization

The conceptual model includes two modules: (a) a simplified population dynamics model, and (b) a landscape-based model for integrating the impact of the pesticide exposure on the population model parameters.

The population dynamics is modelled at an individual level for groups of individuals, “nests”, located in a defined feeding area. Each “nest” is defined by its location, the initial number of individuals distributed in up to four age groups, and the seasonal reproductive period within the year. Each age group has an associated background mortality rate. Females from the reproductive age groups have an associated background reproduction rate. All parameters can be selected and modified by the user, and adjusted to geo-ecological conditions. The modifications allow modelling pesticide effects for populations in expansion, recession, or steady-state conditions.

At daily intervals, the mortality and reproduction rates are used for setting the number of deaths in each age group and the number of new-borns. Deaths are randomly allocated to the individuals in the group, and the sex of the new-borns is also randomly assigned. Then, the age of all individuals is increased by one day (or other selected time period) and those reaching the age-threshold moved to the next age group. The population dynamics can be represented as the evolution of the total number of individuals in each “nest”, the evolution of the distribution of individuals per age group, and the combined distribution of several nests in the area.

The pesticides landscape-based model integrates the impact of the pesticide exposure on the population model parameters for each age-group, as a feeding area around the nest is defined. Each nest is placed in a defined location within the landscape scenario. Each feeding area is then connected to the land use to estimate the percentages for the fraction of the diet obtained for each zone within the feeding area. Following the application of a pesticide, the pesticide concentration in the treated crop and other food items is estimated daily, according to the expected environmental fate. Pesticide concentrations are estimated for each commodity and weighted according to the percentage in the diet. The daily or time-weighted exposures are used as input values for the dose-response curves to estimate the expected impact of the pesticide exposure on the mortality and reproduction rates. These values are selected from the available toxicity data using expert judgement.

#### 2.1.2. Model Parameterization

The population dynamic parameters for each species are: distribution of age groups, background mortality rates, feeding range, and background reproduction rates, and reproductive period. The information for the selected species was retrieved from targeted searchers in PubMed and WebOfScience. The model allows the user to define all these parameters adapting the results to local habitats and regional conditions. The expected variability of these ecological parameters under different habitats and regions was considered for comparative assessments and sensitivity analysis.

The pesticide exposure model implements, at a landscape level, the scenarios of the EFSA guidance for assessing the risk of pesticides to birds and mammals [3]. This guidance is currently under review. The model is based on the last update (guidance published on 26 July 2010) which is mandatory in the EU for regulatory risk assessment of pesticides and will be updated in the future to account for possible revisions. The guidance provides the scenarios, equations, and default values for estimating the level of pesticide residues in the different food items for a large set of crops, and consumption amounts for a set of representative birds and mammals. As the model utterly implements the EFSA exposure assessment for herbivorous mammals, the exposure equations are not duplicated here. The reader is referred to the EFSA guidance [3].

The pesticide effects are modelled through three complementary parameters, that must be selected by expert judgement from the available toxicokinetic and toxicodynamic information:

- Change in acute mortality rate
- Change in chronic mortality rate
- Change in reproduction rate

The extrapolation of the experimental mammalian toxicity data observed in the lab to the expected consequences in the field required several assumptions. In repeated-dose studies, animals are dosed every day at the same level, which is not the case in the field. The change in exposure can be modelled using the time-weighted exposure concentrations [3]; some specific recommendations were provided in the opinion of the EFSA Panel [19] that served as a background for the Guidance. The time required for observing relevant symptoms is usually not considered in regulatory assessments but is essential for modelling population effects. This requires access to the daily or weekly observations for each study; using the time between initiation of the dosing and the observation of the symptoms for setting the length of the period to be used in the time-weighting exposure estimation. Regarding the dose-response curve, the ideal situation is the use of the benchmark dose approach [20], but this is rarely available, and in most cases, only the No Observed Adverse Effect Level (NOAEL) and the Lowest Observed Adverse Effect Level (LOAEL) are available. These parameters are selected from statistical analysis, regardless of the magnitude of the effect, the estimation of the actual magnitude at the time window used for the assessment is problematic in most cases, thus a pragmatic assumption was applied when verifiable quantitative information could not be retrieved from the data, or when the assessment was the result of combining several studies. The default level of effect for the LOAEL was assumed as 25%; the default curve was a linear dose-response, provided that the level of effect for the NOAEL was within the range 5–10%. Linearity was considered appropriate as the relationship models the average effect for the average exposure of each population subgroup per nest, which includes individuals with different exposure levels.

The impact of pesticides on the mortality rates was estimated by combining survival rates according to the following equation:

Accumulated mortality rate = 1 − (1 − Subgroup SR)(1 − Pa SR)(1 − Pb SR)…(1 − Pi SR),(1)

where Subgroup SR is 1 minus the background mortality rate for the population group and Px SR is 1 minus the mortality rate associated to exposure to pesticide x (for x = a to i). Equation (1) is used for estimating the accumulated pesticide impact on the acute and on the chronic mortality. Acute mortalities rates are estimated on daily basis according to the actual exposure value and assuming that there is no background acute mortality. Chronic mortality rates are estimated monthly, considering the background monthly mortality for the population group and time window averaged (TWA) exposure levels. The time for estimating TWA is pesticide specific according to their toxicity profile, the maximum TWA exposure value from the previous month is used for the calculations in Equation (1).

For reproductive effects, both reproduction and developmental studies are considered; combining the relevant observations for maternal toxicity, effects on fertility, and foetal and neonate mortality. In addition to the dose and magnitude of the effect, the time between application and observation of the effects is critical to establish the time period to which the pesticide effect should be applied. For the selected time period, the impact on reproduction was estimated as follows:

Final monthly reproduction rate = (Background rate)(1 − Pa RE)(1 − Pb RE)…(1 − Pi RE),(2)

where the reproduction rate represents the average number of offspring per reproductive female and month during the reproductive window, and Px RE is 1 minus the effect on reproduction associated with pesticide x. The average pregnancy time is considered for selecting the time between exposure and actual observation of the effects on reproduction, i.e., for a pregnancy time of 30 days the effects on the reproductive toxicity rate are observed in the following month. The maximum exposure value from the previous month is used for estimating the effects on mortality and from the two previous months for estimating the effects on reproduction.

#### 2.1.3. Computer Implementation

All calculations were implemented in a computer model developed using the programming language Python version 3. The model was specifically developed for this purpose.

In addition, an executable version with a user-friendly interface was developed. This version offers large flexibility to the user and can be made available under request to potential users for non-commercial purposes. The implemented features include:

- replicability assessment through a number of iterations (within nest replications) selected by the user and expression of results as mean with 95th confidence intervals or maximum/minimum,
- landscape with field areas defined by the user,
- inner and outer adjustable bands for each field, and
- selection of crops, crop rotation and pesticide applications at time dates defined by the user,
- selection of the lagomorph species and location and all ecological model parameters of each nest defined by the user.

The software implements several graphic and tabular outputs for presenting different types of results according to the user’s needs.

### 2.2. Case Study with Lagomorphs

The European rabbit (*Oryctolagus cuniculus*) and the brown hare (*Lepus europaeus*) are the representative species of large herbivorous mammals included in the EFSA guidance [3] and have been implemented for this case study.

The population dynamic parameters were selected from a review of the ecology of these two lagomorph species. Default values and ranges were selected based on information retrieved from targeted searchers in PubMed and WebOfScience for the following parameters: distribution of age groups, background mortality rates, feeding range, and background reproduction rates, and reproductive period.

For this case study, we have selected ecological parameters according to previous reviews representing Mediterranean conditions in the EU. The rabbit reproductive season (six months per year in winter and spring) and rate (average of four litter per month for a reproductive female) are based on the reviews by Tablado and co-workers [21,22] and mortality rates were extracted from reviews from the same authors [21,23]. The brown hare reproductive season in central Europe (seven to eight months per year, covering part of the summer) and rate (average 1.75 litter per month for a reproductive adult female, 0.6 litter per month for a reproductive young female) and mortality rates were extracted from several reviews [24,25,26]. Under Mediterranean conditions, the hare reproductive season may be extended to the full year, but accompanied by a reduction in the litter number [27], resulting in an estimated reproduction rate of around 1 litter per month for a reproductive adult female. Table 1 summarizes the selected default values; different values were used in some model estimations for assessing the influence of population patterns, details are provided in each figure caption.

The third element is the toxicodynamic assessment of each pesticide, selecting the relevant exposure time windows and the dose-response relationships for acute lethality, chronic toxicity, and reproduction. Two herbicides with potential risk for mammals according to the EFSA conclusions, glyphosate [28] and bromoxynil [29], have been used as model chemicals in this study. All studies available on mammals and reported in the supporting information for the EFSA Conclusions are available on the EFSA website and were searched for the toxicodynamic assessment.

## 3. Results

### 3.1. Model Calibration and Validation for Context of Use Qualification

The model simplifies the ecological complexity to estimate the impact of pesticides. It is based on regulatory scenarios and exposure estimations, and consequently is not suitable for a direct field validation. Instead, the model was calibrated and verified for regulatory use qualification by comparing the outcomes of the specific model subroutines (population dynamics, exposure assessment, time-weight estimations, and impact on survival and reproduction rates) with manual calculations using the equations implemented in the model. Following a set of iterative calibrations, a final verification confirmed the consistency between the model and the manual calculations. This was further confirmed with the case study with lagomorphs.

### 3.2. Model Replicability and Flexibility

**Figure 1:**
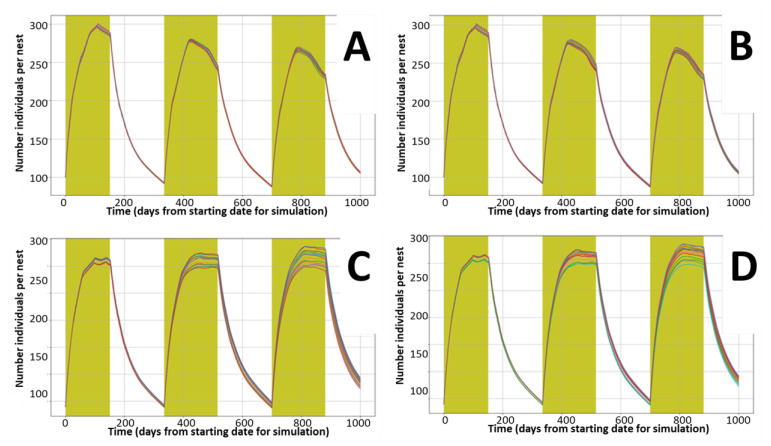
Replicability of total population dynamics (abundance) during a 1000 day period. Each color line represents the average of 50 replications per nest, each graphic presents results for 16 nests with identical properties. (**A**,**B**) Replicates (2 independent runs with same input values) for an initial number of 100 rabbits per nest. (**C**,**D**) Replicates (2 independent runs with same input values) for an initial number of 140 rabbits per nest.

Figure 1 presents the model replicability, investigated through a combination of the iteration tool offered by the model (replication of calculations for the defined nest), parallel running of several nests with identical conditions, and consecutive running of the same conditions several times.

**Figure 2:**
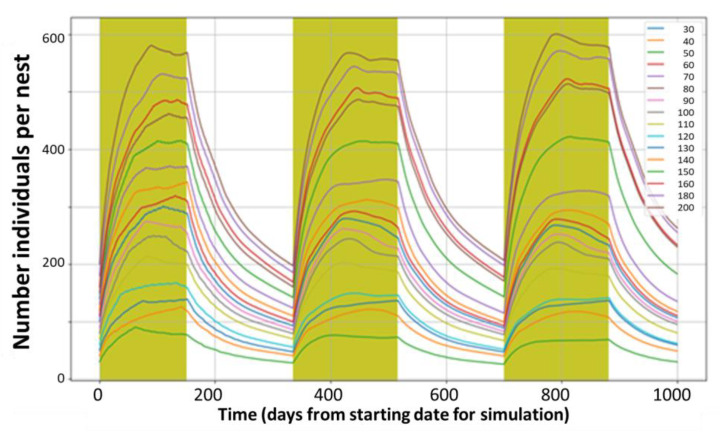
Total population dynamics (abundance) for a period of 1000 days according to the initial number of individuals in the nest (from 30 to 200).

The model reproduces rabbits under Mediterranean conditions (reproduction season from December to June) for starting populations of 100 (A and B) and 140 (C and D) individuals per nest. The observed variability is linked to aleatory allocations of deaths within the age group and sex of new-borns and reflects the expected natural variability. The largest variability is observed during the peak period (end of the reproductive season) for the conditions representing a stable population (C and D). Figure 2 confirms that the proposed default parameters for reproduction and mortality rates represent conditions close to stable populations with large seasonal variability, particularly for initial densities of 140 and 240 rabbits per nest.

**Figure 3:**
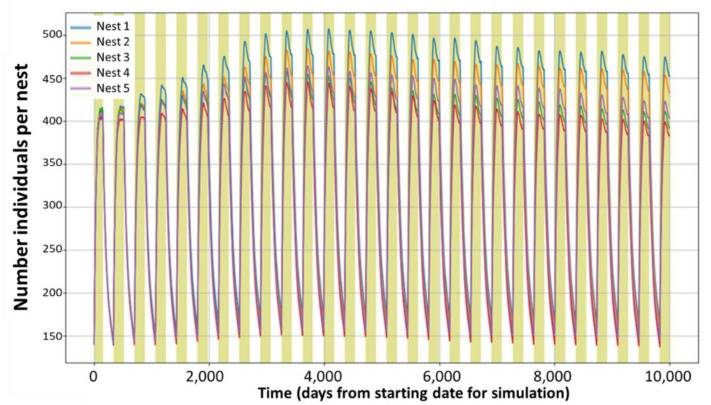
Replicability of total population dynamics during a 10,000 day period. Each line represents the average of 50 replications per nest, with identical properties and an initial number of 140 rabbits per nest.

**Figure 4:**
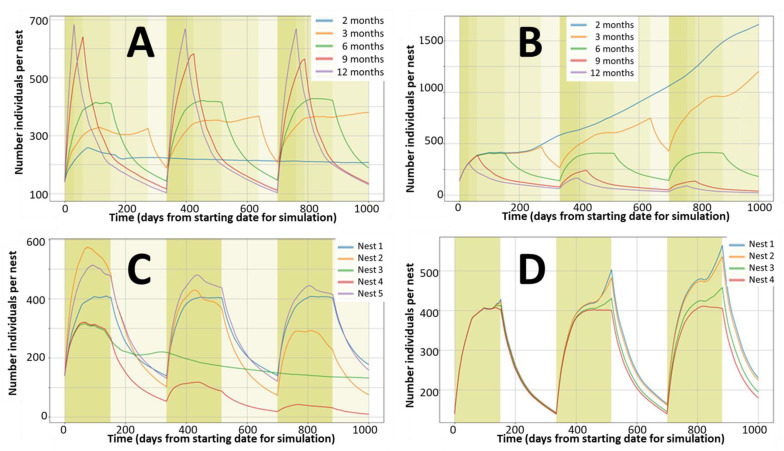
Examples of abundance simulations for different reproduction and mortality conditions for nests with similar initial densities (140 rabbits per nest). (**A**): Reproductive seasons of 2, 3, 6, 9, or 12 months per year with similar adjusted annual reproduction rates. (**B**): Reproductive seasons of 2, 3, 6, 9, or 12 months per year with similar monthly reproduction rates. (**C**): Effects of different combinations for mortality and reproduction (Nest 1 default monthly values of 0.11 for mortality and 4.0 for reproduction with reproduction season of 6 months; Nest 2 rates of 0.22 for mortality and 8.0 for reproduction with reproduction season of 6 months; Nest 3 rates of 0.22 for mortality and 4.0 for reproduction with reproduction season of 12 months; Nest 4 rates of 0.22 for mortality and 4.0 for reproduction with reproduction season of 6 months; Nest 5 rates of 0.16 for mortality and 6.0 for reproduction with reproduction season of 6 months). (**D**): Effect of adding reproductive capacities to the subadult (A2) female group (reproduction rate for A2 of 4.0, 3.0, 2.0, and 0.0 for nests 1, 2, 3, and 4 respectively).

The population peaks at the end of the reproductive seasons, almost triplicating the initial number of individuals and goes back to background levels during the non-reproductive seasons. Figure 3 confirms that under these conditions, the populations are stable for a very long period, while the variability associated with the aleatory allocations increases with time for the first 8–9 years and then stabilises. The model allows the estimation of not stable populations and all kinds of combinations, as presented in Figure 4.

### 3.3. Case Study with Lagomorphs

The toxicological information published by EFSA was used for the data extraction. This information includes the “Conclusion on Pesticides” published in the EFSA Journal and the final Rapporteur Member State assessment with comprehensive summaries of each study published as a background document in the “Registry of Questions” supporting the Conclusion.

#### 3.3.1. Glyphosate

The acute lethality estimation was based on a single dose LD_50_ higher than 2000 mg/kg b.w. and the associated LD_0_ higher than 200 mg/kg b.w. As these values are expressed as “higher than” values, no additional interspecies correction was applied. A linear relationship (linear regression for pairs (200,0) and (2000,0.5) expressing mortality as rates) provides the following equation:

Acute P_glyphosate_ SR = 0.000278ETE_t_ − 0.055556; values below 0 are corrected to 0, (3)

where ETE_t_ represents the average estimated theoretical exposure in mg/kg b.w. of day t for each subpopulation group and nest.

The effect on chronic mortality rate considers LOAELs from subacute oral studies (Section B.6.3.1), long-term toxicity and carcinogenicity (Section B.6.5), and relevant effects from reproductive studies (Section B.6.6). A conservative approach was used in line with regulatory assessments. The lowest LOAEL was selected, including not only mortality but also morbidity under the assumption that in the field these effects will affect the survival capacity of the individual. The selected value was a LOAEL of 175 mg/kg b.w. day for maternal toxicity in developmental studies on rabbits. The selected NOAEL is 50 mg/kg b.w. A default effect value of 25% was allocated to the LOAEL. Although the study is on rabbits, the default interspecies variability factor of 5 for chronic exposures was applied for accounting for differences between the domestic rabbit subspecies *Oryctolagus cuniculus domesticus,* wild rabbits, and hares. Considering the time between initiation of exposure and observation of the effects, a time window of 12 days was fixed for the exposure estimations. A linear relationship (linear regression for pairs (0,0) and (35,0.25) expressing the effects as rates) met the default range effect value (NOAEL ranging between 5 and 10% of effect) and provided the following equation:

Chronic P_glyphosate_ SR = 0.0071ETEtwa_(t−12,t),_ (4)

where ETEtwa_(t−12,t)_ represents the time weight average estimated theoretical exposure in mg/kg b.w. for the 12 previous days to day t for each subpopulation group and nest.

Considering that the maternal toxicity was more sensitive than developmental toxicity, a similar equation covers the reproductive effects:

P_glyphosate_ RE = 0.0071ETEtwa_(t−12,t),_ (5)

where ETEtwa_(t−12,t)_ represents the time weight average estimated theoretical exposure in mg/kg b.w. for the 12 previous days to day t for each subpopulation group and nest.

**Figure 5:**
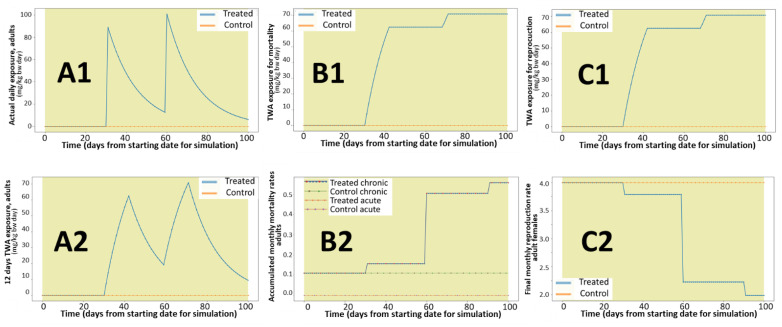
Relationship between daily exposure (**A1**), 12 days time-weighted average exposure (**A2**), the maximum monthly twaETA used for assessing mortality (**B1**) and reproductive (**C1**) effects, and the associated effects on the mortality (**B2**) and reproduction (**C2**) rates, following two applications of glyphosate on cereals at 4.0 kg/ha.

Figure 5 present an example of the evolution of the daily and twa ETEs, the use of the maximum monthly values for the estimations, and the resulting effects on mortality and reproduction rates for two applications of glyphosate at 4.0 kg/ha in cereals during the rabbit reproduction period.

#### 3.3.2. Bromoxynil

The acute lethality estimation was based on a rat single dose LD_50_ of 130 mg/kg b.w. and the associated LD_0_ 10 mg/kg b.w.; with an interspecies uncertainty factor of 5 applied to the LD_50_ but not to the LD_0_ as the lack of mortality is confirmed by other studies in rabbits. A linear relationship (linear regression for pairs (10,0) and (26,0.5) expressing mortality as rates) provides the following equation:

Acute P_bromoxynil_ SR = 0.0313ETE_t_ − 0.3125; values below 0 are corrected to 0, (6)

where ETE_t_ represents the average estimated theoretical exposure in mg/kg b.w. of day t for each subpopulation group and nest.

The effect on chronic mortality rate considers LOAELs from subacute oral studies (Section B.6.3.1), long-term toxicity and carcinogenicity (Section B.6.5), and relevant effects from reproductive studies (Section B.6.6). A conservative approach was used in line with regulatory assessments. The lowest LOAEL was selected, including not only mortality but also morbidity under the assumption that in the field these effects will affect the survival capacity of the individual. The selected value was a LOAEL of 17.1 mg/kg b.w. day from a subacute toxicity study on mice, and the default interspecies variability factor of 5 for chronic exposures. Rats and dogs have lower NOAELs for some sublethal effects but also higher NOAELS in 90 days and 1 year studies. The selected value would be also in line with the benchmark approach proposed in the assessment. Considering the time between initiation of exposure and observation of the effects, a time window of 5 days was fixed for the exposure estimations. A linear relationship (linear regression for pairs (0,0) and (3.42,0.25) expressing the effects as rates) met the default range effect value for the NOAEL and provided the following equation:

Chronic P_bromoxynil_ SR = 0.0731ETEtwa_(t−5,t),_ (7)

where ETEtwa_(t−5,t)_ represents the time weight average estimated theoretical exposure in mg/kg b.w. for the 5 previous days to day t for each subpopulation group and nest.

Regarding reproduction effects, the most sensitive and ecologically relevant observed effect corresponded to a LOAEL of 12.5 mg/kg b.w. day with increased post-implantation loss and malformation, with the default interspecies variability factor of 5 for chronic exposures. Considering the time between initiation of exposure and observation of the effects, a time window of 5 days was fixed for the exposure estimations. A linear relationship (linear regression for pairs (0,0) and (2.5,0.25) expressing the effects as rates) met the default range effect value for the NOAEL and provided the following equation:

P_bromoxynil_ RE = 0.1ETEtwa_(t−5,t),_ (8)

where ETEtwa_(t−5,t)_ represents the time weight average estimated theoretical exposure in mg/kg b.w. for the 5 previous days to day t for each subpopulation group and nest.

**Figure 6:**
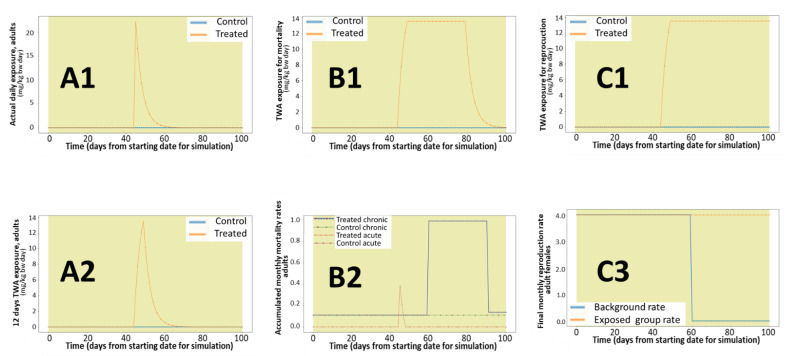
Relationship between daily exposure (**A1**), 5 days, time-weighted average exposure (**A2**), the maximum monthly twaETA used for assessing mortality (**B1**) and reproductive (**C1**) effects, and the associated effects on the mortality (**B2**) and reproduction (**C2**) rates, following one application of bromoxynil on cereals at 1.0 kg/ha.

Figure 6 present an example of the evolution of the daily and twa ETEs, the use of the maximum monthly values for the estimations, and the resulting effects on mortality and reproduction rates for one application of bromoxynil at 1.0 kg/ha in cereals during the rabbit reproduction period.

**Figure 7:**
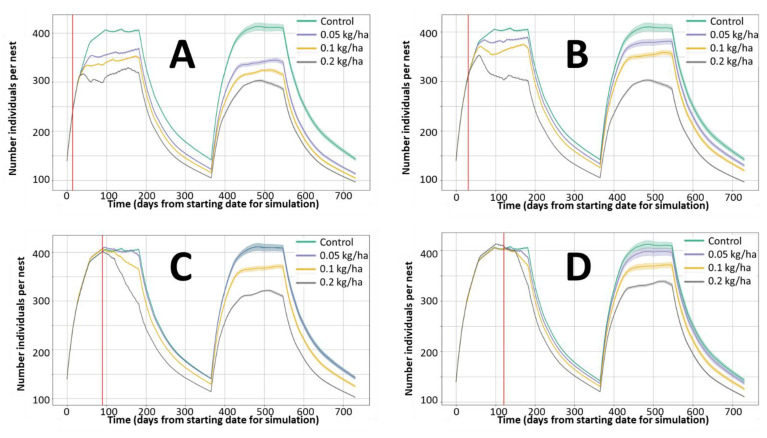
Effect of bromoxynil on the evolution of rabbit abundance (total population number). Nest 1 control; Nest 2 treated 0.05 kg/ha; Nest 3 treated 0.1 kg/ha; Nest 4 treated 0.2 kg/ha. Each figure represents equivalent treatments at different time points (red vertical line) during the breeding season. (**A**): 15 days; (**B**): 30 days, (**C**): 90 days and (**D**): 120 days, after breading season initiation, respectively.

The model allows predictions related to the effect of pesticide applications at a population level. For similar population settings, these effects depend on the toxicity of the pesticide and the application rate, but also on the time of application within the reproductive period. Figure 7 offers an example for the application of bromoxynil at different doses and times for conditions representing a stable population. In line with the information extracted from the EFSA assessment, the exposure to bromoxynil from the treated crop and in-field grass is expected to be a short-term phenomenon, due to the rapid dissipation of the pesticide residues. If the acute lethality threshold is exceeded, some impact may be observed immediately, while most of the effects on the population dynamics will be delayed for several weeks accounting for effects on the mortality rate associated with chronic morbidity and on the reproduction of exposed females. The effects of application rates as low as 0.05 kg/ha of bromoxynil are visualised for applications occurring at the beginning of the reproductive season, but are least evident or even disappear for later applications. In the case of very late applications, only minor effects are observed within the season even at 0.2 kg/ha, but the consequences are very clear in the next season even without additional pesticide applications.

**Figure 8:**
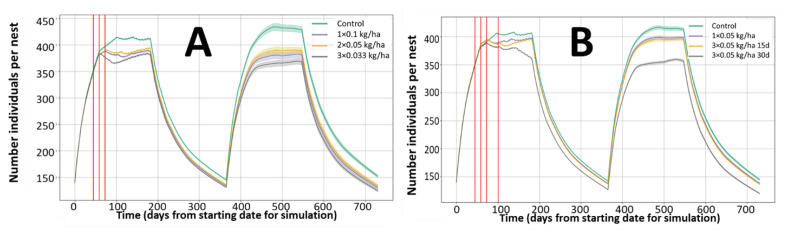
Effect of multiple applications of bromoxynil on the evolution of rabbit abundance (total population number). (**A**): One, two, or three applications summing 0.1 kg/ha. Nest 1 control; Nest 2 One application 0.1 kg/ha; Nest 3 two applications of 0.05 kg/ha; Nest 4 three applications of 0.033 kg/ha. (**B**): Effect of application time: Nest 1 control; Nest 2 One application 0.05 kg/ha; Nest 3 three applications of 0.05 kg/ha at 15 days intervals; Nest 4 three applications of 0.05 kg/ha at monthly intervals.

The decision on the timings selected for connecting the observed experimental results with the expected impacts on rates in the field is also relevant for assessing multiple treatments. The proposal selected for glyphosate and bromoxynil in these estimations is to link max twaETE observed in a month with the rates of the following month. This approach allows for the generic estimations intended for a simplified model, as the actual breeding season will change, but should be considered by the risk assessors in the assessment of multiple applications as exemplified in Figure 8. As fixed month intervals are selected for the monthly rate adjustment if the pesticide dissipation half-life is much shorter than the interval during treatments, the selection of the actual application dates will change the maximum monthly twaETAs and consequently the impact on the rates and population abundance. These differences should be included in the sensitivity analysis.

The EFSA guidance considers the brown hare as the focal species for grassland and vineyards. Figure S1 in the Supplementary Material simulates the combined effect of two treatments (end of winter and spring/summer) on a stable brown hare population in central Europe (see Table 1 for details). The results confirm the need for assessing the combined effects of different applications through the season in population-based risk assessments.

## 4. Discussion

The examples provided in Figure 5 and Figure 6 indicate the expected exposure estimation (based on EFSA guidance equations [3]) and how this exposure is translated into effects on survival and reproduction rates according to the equations implemented. These effects are implemented as impacts on the population dynamics in Figure 7 and Figure 8. To facilitate the verification of the results, these examples assume maximum exposure from the treated field according to work-cased conditions of the EFSA guidance. The landscape component of the model allows exposure from different fields, crops, and crop rotations according to the user’s design.

Topping et al. [30] have addressed the shortcomings of current pesticide environmental risk assessment strategies and suggested an integrated system approach. The proposal implements opinions from the EFSA Panels and includes better use of available ecotoxicity data, the incorporation of landscape tools, and the need for integrating the risks from different pesticides, stressing the limitation of the single product and single crop approach [30].

The ecotoxicity assessment of wild mammals is rarely based on dedicated ecotoxicity studies. Generally, the assessment is based on the re-evaluation of the safety human health studies. This has the benefit of a relatively large number of studies with different designs, as required to cover the human hazard assessment, but with the limitations of studies and endpoints targeted to humans. As confirmed by the glyphosate and bromoxynil examples, instead of focusing on selecting the lowest relevant NOEL as the Reference Point, the information can be evaluated from a different perspective and used to establish dose-time-response relationships. The model addresses the impact of pesticides on mammalian populations [30] by combining all morbidity effects into the combined effect on the monthly mortality rate. The interpretation of the time patterns from the toxicological studies is a critical element for assessing the effects of pesticides. The information available is based on standard toxicity testing for assessing repeated dose effects with continuous dosing. This situation requires the reanalysis of the daily data from each test to identify the time required for observing the effects. The comparison of the NOAELs and LOAELs from studies with different duration may offer relevant information [31]. For glyphosate and bromoxynil, the lowest LOAELs were observed already at short exposure durations, suggesting the use of exposure time windows of 12 and 5 days for glyphosate and bromoxynil, respectively. Achieving the maximum toxicity level within a relatively short time period is a frequent feature in chronic toxicity testing, observed for about 50% of pesticides [32] as well as chemicals in general [33], being more frequent for chemicals with high toxicity [31]. Based on this information, substance and endpoint specific values are required regarding the number of days to be used for estimating the time-weighted average exposure.

While standard risk assessment approaches focus on setting thresholds, our proposal for population modelling is to use the full dose-response curve and to integrate the time component, selecting for each pesticide the relevant duration of effects associated with chronic exposure according to the information extracted from the different studies. This is in line with the use in ecotoxicology of novel dose-response tools such as the benchmark dose approach [31]; it also considers that at the population level, the pesticide exposure will vary both over time and among individuals, and consequently the effect on mortality and reproduction rates is better modelled by a continuous rather than a binary response. Extracting information from the toxicity studies and constructing the dose-time-response models requires expert knowledge and has an associated level of uncertainty. In addition to general tools for reducing expert bias such as expert knowledge elicitation [32], the advantage of modelling versus testing is the possibility for conducting sets of assessments covering the variability of the different parameters and presenting sensitivity analysis estimations.

In line with the EFSA Scientific Committee and PPR Panel [2,33] specific protection goals linked to the provision of ecosystem services can be established through five complementary dimensions: ecological entity, attribute, magnitude, temporal, and spatial scales. For wild mammals, the ecological entity is the population, and the most relevant attributes of survival and reproduction. Our model provides a tool for assessing the magnitude of the expected effects on these attributes and their temporal and spatial scale. In addition, these models offer the possibility for implementing in practice the “ecological recovery option” [34,35], which is proposed in the EFSA guidance for aquatic organisms [36], but not implemented for terrestrial vertebrates yet. Seasonal recovery is directly estimated by the model and results from the combination of the duration of the reproductive season and the background reproductive rate. The estimations presented in Figure 1, Figure 2, Figure 3, Figure 4, Figure 5, Figure 6, Figure 7 and Figure 8 assume that the background reproductive and mortality rates are independent of the number of animals per nest; this is a simplification as for most animal species population’s growth rate decreases with animal density [37]. The use of the recovery option requires the inclusion of this dependency in the rate equations; from the modelling perspective, this inclusion can be easily incorporated once the dependency is clarified. However, from the ecological perspective, quantifying this dependency requires specific local information elements on factors, triggering the link between population density and population dynamics, such as predation pressure, competition on habitat, and availability of food. In fact, recovery may be relevant under some conditions but not under others.

In regulatory risk assessments, assessing the effects of single or multiple applications of a pesticide on a specific crop and field is standard practice. However, the combination of effects from treatments in different fields and crops is currently lacking. The inclusion of this possibility is essential for ensuring that the risk characterisation results are informative and can be considered in environmental impact assessments [14]. Most approaches for assessing the effect of pesticide mixtures focus on mixture toxicity principles [38], assuming co-exposure. Significant research in this area is on-going [39]; however, in landscape modelling at the population level, in addition to the same individual being exposed to several pesticides, the need for combining effects of different individuals nesting on different crops is also very relevant. Offering this capacity is a key feature of this simplified model.

Predictive models reproduce sets of patterns observed at different scales and levels; the more patterns a model can reproduce simultaneously, the more reliably it may capture the essential features of a real system’s organization; however if a model is too complex, its analysis will be difficult to interpret; thus there is a need for finding the optimal zone of model complexity, the so-called “Medawar zone” [40]. For regulatory use, the level of complexity should be adapted to the “regulatory question”. In our model this need is covered by offering the user the capacity to decide the level of complexity adequate for each problem formulation; e.g., from a simplified landscape (single crop and edge of crop scenario), single pesticide and default parameters; to a complex landscape simulating realistic local conditions, an unlimited number of pesticides and applications, and ecological parameters adapted to actual conditions and environmental pressures. The landscape is selected by the user, based on fields specified by polygons. For local and regional assessments, the real landscape structure can be reproduced by the model, e.g., imported from GIS databases, such as those developed in the EU for implementing the Common Agricultural Practice. A simplified model with four rectangular fields is suggested for generic assessments, but the landscape can be defined in complex polygons resembling an actual or hypothetical location. The crop and crop rotation are defined for each field. The user also defines, for each crop, the pesticides and their application patterns; defining the day of application, the application rate, and the edge-of-field drift according to the intended risk management options. The nests can be located within or outside the fields. The feeding area for each group is directly estimated by the model, as well as the average twaETE according to the EFSA guidance calculations. A key element of this model is that it can refine current risk assessments using only existing standard regulatory toxicity test results. This is a key feature for population models to be used in the regulatory context [41].

There are clear challenges for incorporating higher tier data in the risk assessment of pesticides [42]. The use of the model offers a set of benefits. The exposure to and the impact of pesticides is highly dependent on the landscape agroecological conditions, and models allow the assessment of this variability. Models can also identify the most sensitive factors and prioritize further data collection and refinement [43]; this is also implemented in this model, which supports tiered approaches, moving from simple to complex assessments, which is a key principle for regulatory risk assessments.

## 5. Conclusions

Although the use of population models has been identified as a clear opportunity, the incorporation of ecological models in the regulatory context is still minimal, and most examples focus on the aquatic compartment. Based on the EFSA, good modelling practices, and other international recommendations we have developed a simplified, flexible, and versatile population model for assessing the risk of pesticides to herbivorous mammals. This landscape model combines (a) regulatory exposure scenarios, (b) standard regulatory toxicity results, and (c) ecological principles, to model the effect of pesticides on the population dynamics of rabbits and hares. The model reproduces simplified and realistic agricultural landscapes, considers crop rotation, and can run estimations for single and multiple applications of one or several pesticides per field. Background reproduction and mortality conditions (seasonality and rates) can be modified by the user and adjusted for each sub-population group (nest). A case study with estimations for two pesticides glyphosate and bromoxynil, confirms the capacity of the model for identifying the risk drivers for pesticide applications under different ecological and landscape conditions.

## Figures and Tables

**Table 1:** Summary of default values used for the model estimations presented in the figures. Deviations from these values are indicated in each figure caption.

Parameter	Rabbit	Brown Hare
Number of age groups number	4 (0,1,2,3)	4 (0,1,2,3)
Background monthly mortality rate (groups 0/1/2/3)	0.77/0.38/0.11/0.11	0.87/0.30/0.08/0.08
Background monthly reproduction rate (groups 0/1/2/3)	0/0/0/4	0/0/0.6/1.75
Reproductive season	December to May	January to August
Initial number of individuals per nest	140	100
Initial male/female rate	1:1	1:1
Parameters for exposure estimation	As by EFSA guidance (EFSA, 2009)	
TWAexposure-effect relationships	Equations developed for this study following the review of available information in the EFSA Conclusions	

**Figure A1:**
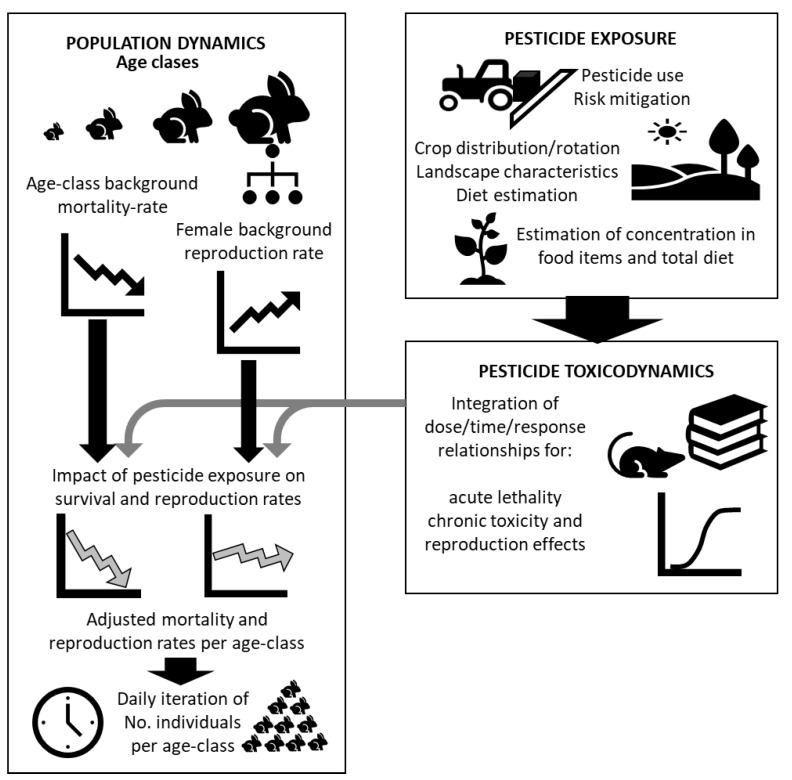
Representation of the conceptual model.

## Data Availability

All data used for the model calculations are publicly available at the cited references or at the EFSA webpage (www.efsa.europa.eu, (accessed on 13 December 2020)). An executable version with a user-friendly interface was developed. This version offers large flexibility to the user. The code and the executable version may be available under request to potential users interested in checking the usability of the model, with no cost for non-commercial purposes.

## Supplementary Materials

The following are available online at https://www.mdpi.com/article/10.3390/ijerph18157720/s1, Figure S1: Effect of two herbicide treatments in vineyards (end of winter and spring/summer) on the evolution of brown hare abundance (total population number).

## Appendix A

Description of model characteristics according to the EFSA scheme on Good Modelling Practice [11].

### Appendix A.1. Problem Definition

The model addresses landscape variability in the pesticide risk assessment process. The focus is on terrestrial vertebrates and the aim is the identification of the key risk drivers impacting on mid-term population dynamics. The model has been developed implementing species parameters and pesticide exposure models of the EFSA Guidance Document, but can be adapted to other regulatory schemes. The population dynamics is modelled through daily survival and seasonal reproductions rates, which are modified in case of pesticide exposure. All variables, parameters, and functions can be modified.

### Appendix A.2. Supporting Data

The pesticide exposure assessment implements the crops, focal species, and default values of the EFSA Guidance document.

For this publication, focusing on the large herbivorous comparative model, the survival and reproduction parameters for rabbits and hare have been selected following a literature search on wild rabbits and hare population ecology.

The functions describing the impact of pesticide residues on survival and reproduction rates have been estimated using expert judgement following the in-depth assessment of the Risk Assessment Reports published as background documents by EFSA supporting its Conclusions. For this publication, two pesticide active substances, Glyphosate and Bromoxinyl, have been used as model pesticides.

### Appendix A.3. Conceptual Model

The conceptual model includes two modules: (a) the species population dynamics, and (b) the impact of the pesticide exposure, which includes two submodules, one to estimate the concentration in the food items and total diet considering the landscape characteristics and the toxicodynamic assessments for selecting the relevant exposure time windows for the chronic effects and the dose-response relationships for acute lethality, chronic toxicity, and reproduction. Figure A1 presents the conceptual model diagram.

The population dynamics model is based on daily iterations of survival, per age class, and reproduction, for females in reproductive age and during the seasonal reproduction period. Background mortality and reproduction rates are adjusted to geo-ecological conditions and can be adjusted for representing populations in expansion, recession, or steady-state conditions.

The pesticide exposure model implements the EFSA guidance scenario for a simplified landscape scenario, based on adjustable land-use models, allowing for crop rotation.

The more complex aspect of the model, requiring expert judgement, is the toxicodynamic assessment. This assessment requires the revision of all available experimental and field studies available. The EU assessments consist of the EFSA Conclusion, and a detailed Risk Assessment document drafted by the Rapporteur Member State and peer-reviewed by EU experts during the EFSA process. The dossier submitted by the applicant is also available. This information offers a sufficient level of detail for extracting the information to be used for adjusting the mortality and reproduction rates to different levels of pesticide exposure. Information, although with different levels of detail, is also published by other jurisdictions world-wide, including the US EPA Office of Pesticides Programs, the Health Canada Pest Management Regulatory Agency, the Australian Pesticides and Veterinary Medicines Authority, or the Food and Agricultural Materials Inspection Center in Japan.

Animals are grouped in nests, and the population dynamics is estimated for each nest, which can also be grouped for several nests. The number, sex, and age of each individual in the nest is estimated with daily iterations, adjusted by the associated survival rate for each group, the number of new-borns during the reproductive period, and pass to the next age-class when reaching the selected threshold. There are random allocations for mortality within the group and the sex of new-borns, providing environmental variability to the model estimation.

For each age-group, a feeding area around the nest is defined. Each nest is placed in a defined location within the landscape scenario. Each feeding area is then connected to the land use to estimate the percentages for the fraction of the diet obtained for each zone within the feeding area. Following the application of a pesticide, the pesticide concentration in the treated crop and other food items is estimated daily, according to the expected environmental fate. Pesticide concentrations are estimated for each commodity and weighted according to the percentage in the diet. The daily or time-weighted exposures are used as input values for the dose-response curves to estimate the expected impact of the pesticide exposure on the mortality and reproduction rates.

### Appendix A.4. Formal Model

The model variables for the different modules are summarised below.

Landscape variables:

Total area. Defined by the parcels defined by the user, accommodating any actual size.

Land sectors. Number, form, and location defined by the user. Each sector (field) includes:

- Location and form (polygons) are defined by setting coordinates for the vertices.
- Associated crop, or a different land use (e.g., grassland, bare soil, forest), defined by the user, which can be changed to represent crop rotation.
- Associated inner and outer edge. The inner edge allows setting in-field risk mitigation options, such as uncropped or unsprayed buffer zones. The outer edge is used for estimating pesticide contamination due to spry drift in adjacent areas. The edges can be selected for each land sector.
- Residual concentration of pesticides at time zero, allowing the consideration of residues from previous exposures
- Pesticide applications, the number is defined by the user and each application is defined by:
  ○ Pesticide
  ○ Date of application
  ○ Dose
  ○ Interception, to estimate the dose that reaches the feeding items for the species
  ○ Dose ratio for the inner edge, to account for unsprayed
  ○ Dose ratio for the outer edge

A default four-land scenario, distributing the land in four equal squares, is available and allows most estimations for regulatory purposes. Specific local scenarios can be easily included allowing GIS modelling.

Population dynamics is modelled through groups of animals or “nests” defined by the following variables:

- Species
- Location, defined by coordinates
- Initial population, defined by a number of individuals, average age, and age standard deviation
- Breeding months
- Four age groups, with defined age thresholds and food commodities, and the following associated variables that can be modified by the user:
  ○ Initial male/female ratio
  ○ Age range
  ○ Average mobility range
  ○ Maximum mobility range
  ○ Background mortality rate for males
  ○ Background mortality rate for females
  ○ Background reproduction rate
  ○ Attractiveness factor for the associated food commodities.

The toxicodynamic parameters to be established for each pesticide are:

- Time variables: Use of actual or time-weighted averages for the pesticide exposure, and the averaging period when relevant.
- The exposure-response curves, for acute mortality, chronic mortality, and reproduction
- The time-scheme for updating the chronic mortality and reproduction rates

The pesticide exposure estimations are based on the parameters and equations of the EFSA Guidance.

### Appendix A.5. Computer Model

The model was specifically developed for this purpose. The model was implemented using the programming language Python version 3. In addition, an executable version with a user-friendly interface was developed. Errors, bugs, and inconsistencies in the code were analysed through a series of examples covering all possible options.

A set of examples were conducted for each selected pesticide. Pesticide exposure and the estimated effect on the reproduction and mortality rates were calculated through the model and confirmed through a parallel estimation of exposure and effects estimations using the proposed equations. The errors were corrected, and the examples recalculated until the model and manual estimations provided the same values for all examples and scenarios.

### Appendix A.6. Regulatory Model—The Environmental Scenario

The model has been developed as a supportive tool for prospective and retrospective environmental risk and impact assessments related to the use of pesticides for crop protection. Prospective uses include those supporting the decision-making process in the context of regulatory pre-marketing and re-evaluation assessments (i.e., linked to the process for authorising the use and setting use conditions and risk mitigation options for single pesticides active substances and plant protection products), comparative assessments (e.g., for selecting the less risky alternatives in integrated pest management programs), and for assessing the combined impacts of several plant protection products used in the same area through the season, a year, or a multiannual period. Retrospective uses are linked to monitoring programs, once the model parameters have been adapted to the landscape and ecological conditions, the model may provide estimations on the ecological pressure expected for the actual pesticide use and comparative assessments on the role of the different stressors.

The model focuses on the agro-ecosystems, defining the landscape according to crop distribution, crop management, and crop rotation, and is linked to the use of pesticides in these areas.

### Appendix A.7. Regulatory Model—Parameter Estimation

The model implements the scenarios of the focal species described in the EFSA GD, i.e., rabbits and brown hares as focal large herbivorous species. The food items are associated with the crop, the consumption is based on energetic needs and Food Intake Ratios per body weight mass. Pesticide concentrations in food items are based on the Residue per Unit Dose, and the Expected Theoretical Exposure is estimated for the average consumption of food items and related pesticide concentration per age group.

The ecological parameters for the species to be modelled are described in Section 4, such as age-class thresholds, initial number of individuals by nest, background rates for mortality and reproduction, breeding period, etc., can be extracted from general publications on the ecology of the species and selected by the user according to the specific needs. It should be highlighted that the same species may have very different values for these parameters, thus this flexibility allows the user to adapt the values to specific needs, e.g., in case of assessment for a particular zone, country, or region, or to include extreme values for conducting a sensitivity analysis (see Section Appendix A.8). In addition, the model can be run using different values for each parameter in order to detect the risk triggers (elements that have a higher impact on the final risk characterisation outcome). Generic values instead of values for pre-selected species may be used to determine the ecological characteristics of the most vulnerable species (i.e., ecological vulnerability traits).

### Appendix A.8. Regulatory Model—Sensitivity and Uncertainty Analysis

The sensitivity analysis should be performed for each problem formulation. The model has large flexibility, allowing the user to select the values for the critical parameters and to compare the results at different levels in the risk assessment process: exposure to one or several pesticides for each age-class; hazard characterisation as a modification of survival and reproduction rates; and risk characterisation through the direct comparison of the population dynamics.

The uncertainty assessment for the model parameters with random allocation (i.e., allocation of the ecological rates to each individual in the age-group and sex) is incorporated by the module that allows the user to run the model several times selecting the number of iterations and to aggregate the results, presenting the 95% confident intervals and the maximum and minimum values in addition to the average value.

The model has been intentionally designed as a simplified tool using averaged values per nest (i.e., group of animals in the same location) this is specifically important for nests getting food from several crops/areas and being exposed to more than one application (for the same or different pesticides). Average values for the next instead of a threshold approach are appropriate in this context. If needed, uncertainty and variability may be addressed by running the model for the individual exposure levels and combining the results.

### Appendix A.9. Regulatory Model—Comparison with Measurements

The results of all intermediate steps, e.g., pesticide concentration in the different diet commodities, overall pesticide exposure, and impact of pesticide exposure on the rates have been confirmed by running in parallel a large set of scenarios in the model and conducting manual estimations with the same equations and values for the relevant parameters. For the exposure assessment, the selected scenarios were extracted from the EFSA GD, confirmed that the exposure estimation from the model complies with the exposure to be estimated by using the EFSA GD.

### Appendix A.10. Reality/Problem—MODEL Use

The details presented for the problem formulation are described in the main text of the publication.

### Appendix A.11. Reality/Problem—Conclusion

The conclusions for the selected problem formulation are described in the publication. The model is a tool and provides quantitative estimations for the expected population dynamics, the user should compare the results with the relevant protection goals and levels of acceptability for the impact.
