# Supplementary material for: A Simplified Population-Level Landscape Model Identifying Ecological Risk Drivers of Pesticide Applications, Part One: Case Study for Large Herbivorous Mammals
Source: Int J Environ Res Public Health. 2021 Jul 21;18(15):7720. doi: 10.3390/ijerph18157720 (PMC8345457; doi:10.3390/ijerph18157720)
Supplement: Supplementary file 1 [file ijerph-18-07720-s001.zip › ijerph-1285425-SI.pdf]

A Simplified Population-Level Landscape Model Identifying Ecological Risk Drivers of Pesticide Applications, Part One: Case Study for Large Herbivorous Mammals.

David Tarazona, Guillermo Tarazona, Jose V. Tarazona*

* Corresponding author. Email jose.tarazona@efsa.europa.eu

Supplementary material

Simplified simulation for brown hare. In the EFSA guidance, brown hare is the focal species for applications in grassland and vineyards. Typical uses of herbicides in vineyards for controlling weeds when needed include two applications, one at end of winter and one in spring/summer. Simulations are provided for the two selected herbicides at different doses and combinations, the simulation only intends to demonstrate the capacity of the model, and do not represent use doses authorised or typical applied to this crop.

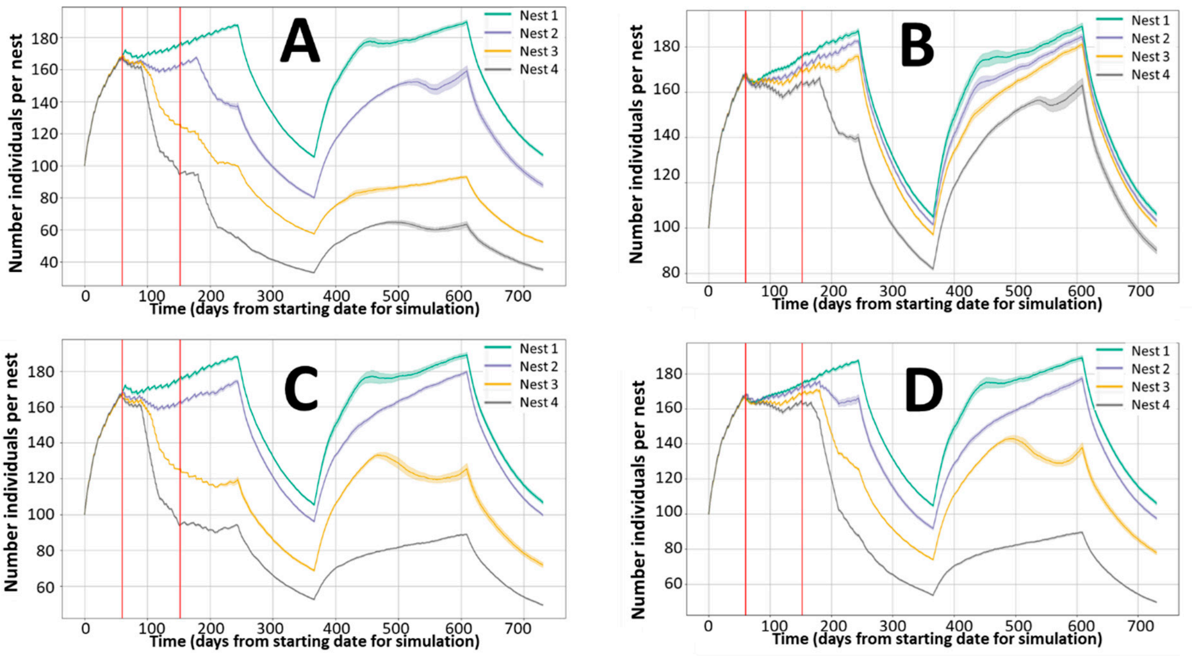

Figure S1. Effect of two herbicide treatments in vineyards (end of winter and spring/summer) on the evolution of brown hare abundance (total population number). Nest 1 control; Nest 2 low dose (glyphosate 2 kg/ha; bromoxynil 0.05 kg/ha; Nest 3 medium dose (glyphosate 4 kg/ha; bromoxynil 0.1 kg/ha); Nest 4 high dose (glyphosate 8 kg/ha; bromoxynil 0.2. Each figure represents different combinations: A two glyphosate treatments, B two bromoxynil treatments, C first glyphosate and then bromoxynil, D, first bromoxynil and then glyphosate.
